# Effect of the Flipped Classroom and Gamification Methods in the Development of a Didactic Unit on Healthy Habits and Diet in Primary Education

**DOI:** 10.3390/nu12082210

**Published:** 2020-07-24

**Authors:** Gerardo Gómez-García, José Antonio Marín-Marín, José-María Romero-Rodríguez, Magdalena Ramos Navas-Parejo, Carmen Rodríguez Jiménez

**Affiliations:** Department of Didactics and Scholar Organization, University of Granada, 18071 Granada, Spain; gomezgarcia@ugr.es (G.G.-G.); jmarin@ugr.es (J.A.M.-M.); magdalena@ugr.es (M.R.N.-P.); carmenrj@ugr.es (C.R.J.)

**Keywords:** nutritional education, educational innovation, Flipped Classroom, gamification, primary education

## Abstract

Currently, there are several methodological models that have broken into different disciplines of knowledge with the aim of making the teaching/learning process more dynamic, active and participatory for students. This is the case of Flipped Classroom, which is based on a mixed approach between e-learning and face-to-face teaching, as well as gamification, which bases its didactic principles on the recreational components of the games. Within this context, the aim of this research is to observe what effect the application of Flipped Classroom and gamification has in the development of motivation, autonomy and self-regulation towards learning through a didactic unit on healthy habits and diet in 202 students of 6th grade of Primary School from four different schools (public and state-subsidized) in the city of Granada (Spain). For this purpose, a methodological design was used with pre-test and post-test to check the effects of the experience on the students. The findings obtained showed that the application of these methods promoted an increase in students’ motivation, as well as in their autonomy and self-regulation when facing the contents of the subject. For this reason, it is advocated that there is a need to continue promoting a quality and innovative educational practice according to the figure of the student today.

## 1. Introduction

People’s eating habits are acquired during their youth and these are conditioned depending on personal tastes and the aesthetic aspects of each individual [1]. However, at this stage of life, ignorance of various topics, including nutrition and correct eating habits, also converges with this acquisition of habits [2]. All of this means that in many cases there is a negative influence on the state of health of people and that this lack of knowledge or nutritional education can lead to diet-related health problems such as obesity, anorexia or bulimia [3,4].

The lifestyle changes experienced during this time of youth bring many new elements, both negative and positive. The changes and acquisition of eating habits that occur are accompanied by concerns about aesthetics, physicality, weight and lack of knowledge about everything related to nutrition. Therefore, in the stage in which there is, on average, the highest intake of nutrients, it is necessary that this knowledge is given in an appropriate way to avoid and prevent nutritional deficiencies [5]. Nutrition education reduces the risk of future diet-related diseases by shaping eating and lifestyle behaviours early [6].

If this nutritional education is not given in an adequate way, there are multiple physical and psychological problems that can become associated with it, like obesity among many others. Thus, obesity is a disease that can have different origins and is always caused in the stages of childhood or adolescence [7]. At the age of these two stages, it is very difficult to treat, since any nutritional imbalance affects the growth of the child [8]. Effective nutrition education implies the involvement of the entire educational community, learning by doing or the creation of different resources or specific tools to achieve a change in eating behaviour [9].

In this respect, technology can play a fundamental role, since through active participation with simulations in the school environment, not only is different knowledge acquired, but also diet and eating habits are experienced in a personalized way, generating a more real awareness than through traditional teaching [10].

### 1.1. The Methodological Approaches Flipped Classroom and Gamification

The educational context and all its agents have the need and obligation to equip themselves with the capacity to analyse and research new methodological strategies that give a specific response to each student and their individual characteristics [11].

The idea is to offer students the ability to learn in an autonomous way, making responsible use of different learnings. The objective is to provide them with skills to be able to identify, evaluate, manage and summarize information in order to confidently face the demands of the society in which they find themselves [12].

The game has been throughout history linked to the culture of the human being [13]. Therefore, employing the game as a learning methodology is a derivation to the educational sphere. This strategy has been proven to be effective when teaching new theoretical and practical knowledge, as well as for the reinforcement of others already acquired [14]. Aspects such as problem solving, collaboration and communication, content creation, among many other things, are worked on through the game in an active way and this methodological strategy has recently been called gamification [15].

This is a recent term; authors such as [16] place its appearance in 2008, although there was no adoption of the term until 2010. Following different complementary definitions of the concept of gamification [13,16], it is about the application of the principles of video games applied to the process of Teaching-Learning (E-Learning), that is, in contexts not strictly related to the game, through the establishment of challenges that, once passed, provide a reward directly proportional to the difficulty of the challenge [17]. It is important to note that the concept of gamification is different from that of an educational game or a game itself [18].

Specifically, gamification refers to the introduction of playful elements to develop learning [19]. Currently, one of the most widespread gamification systems in the educational community is gamification through badges [20]. Thus, the student who obtains different badges is a symbol that they have managed to perform a task or activity satisfactorily, or have had positive behaviour in class [21].

The advantages that this strategy brings are as follows:

- The use of time and performance are increased and more efficient [22].
- Significant increase in motivation and interest in and commitment to the content or subject matter taught [23].
- Collaborative work is promoted [24].
- Diversity is catered for, respecting the particular times and ways of learning, as well as the individual characteristics of each student [25].

As seen in this methodological strategy, technology may or may not be included, depending on how you approach it in particular. However, as seen in education research in recent years, the trend is to incorporate Information and Communication Technologies (ICT) into the teaching/learning processes [26,27,28].

From this incorporation of multimedia devices and new trends arises the Flipped Classroom or Inverted Classroom [29]. In recent years, this methodology is becoming consolidated as an upward educational trend [30,31].

This method is defined as an exchange of educational roles, meaning that now the teacher is the one who acquires a secondary role by being a guide in the learning process, while the student is the one who learns a content outside the classroom, so the tasks traditionally developed at home and in the classroom change; the content is visualized in non-formal contexts and within the classroom, doubts are solved and work is done on this content, either individually or in collaborative groups, making learning active and focused on the student [32,33].

The Flipped Classroom is, by definition, a two-phase methodology. The first phase involves the acquisition of content through the visualization of this through the materials provided by the teacher; these materials are multimedia, such as videos and podcasts, among others [34]. The second phase is developed in the classroom, where students perform interactive readings, problem solving, experiments, role-play, creations, designs, etc., all focused on the subject or theme being worked on at that time [35].

The advantages of the implementation of this methodology within the classroom are as follows:

- Increased motivation in students [36,37,38,39].
- Increased self-perception and self-regulation of learning [40,41].
- Enhanced collaborative work [42].
- Positive increase of academic results [43,44,45,46].
- Giving responsibility to the student, who is aware of his/her own learning process, and increases his/her capacity for autonomous learning [47,48].

Table 1 below summarizes the pedagogical principles behind the two methodologies described, as compared to traditional teaching.

### 1.2. State of Art

After all the above, it has become clear how these methodologies and methodological strategies are widely used in different areas of knowledge.

In general, traditional methodologies assume a passive role of the student, where participation on his part is scarce and his role is limited to being a receiver of the knowledge transmitted by the teacher [49]. Thus, the use of innovative methodologies or methodological strategies related to technology implies an increase in student motivation as well as improvements in performance, interest, grades, teamwork, etc. [50,51].

Thus, it is easy to find research carried out in recent years that aims to demonstrate what the combination of these methodologies entails along with the learning or practice of certain skill concepts in educational centres. These educational centres may be of different types, i.e., public or private, which, depending on the variable being studied, may lead to different results or nuances. With regard to ICT and the methodologies that use them, several studies have shown that there is no significant difference in the implementation of ICT and the acquisition of skills through them, regardless of the character of the school [52,53].

Thus, Gilboy et al. [54] carried out an investigation of the students of the nutrition degree, putting into practice the Flipped Classroom methodology in the subjects taught in that degree. After this experience, it was observed how the students were more committed to their learning; once students understood the causes of this new way of working, there were no noticeable problems. Similarly, there was a minimization of distractions and a lack of interest in conferences or virtual talks, as they were now seen from home and in shorter time slots.

On the other hand, McEvoy et al. [55] carried out an experience with primary education students, where medical students using Flipped Classroom transmitted to this group basic content on nutrition and eating habits. The results showed how this knowledge was widely acquired and, furthermore, the teachers saw this way of teaching content as something feasible and recommendable. Along the same lines, Yildiz et al. [56] carried out a study that related ICT, the skills of 21st century students and crosscutting themes such as nutrition and the promotion of healthy lifestyle habits, all within the framework of transdisciplinary projects within the curriculum.

The combination of both learning strategies has been used in several studies [57], which show that the combination of both techniques not only brings the sum of the benefits of both, but also eliminates the disadvantages that may occur, such as the disconnection of students at home regarding the Flipped Classroom.

There are many studies that deal with nutritional issues, both in specific subjects or careers and in cross-cutting themes present in training at all stages, and which are not only related to this inverted classroom methodology, but also to other current ones such as mobile learning [58] or to the design of specific materials that include all of the above [59].

Thus, there is another large amount of research that continues to relate technology and play with nutrition, diet and decisions about one’s own health, all of which is approached from the perspective of gamification within the classroom [60]. This methodological strategy strengthens one’s awareness of healthy lifestyle habits in daily life, increases the concept of responsibility for the subject and modifies or changes certain unconsciously acquired behaviours [61].

Current studies such as [62] advocate for the use of innovative applications or methodologies through technology, demonstrating that nutrition education is possible that is direct, personalized [63] and instantaneous [64], and that provides assurance about what is being done and how [65].

Nutritional education requires the arrival of new methodological approaches that will make the teaching processes dynamic (especially evaluation) and that will make this subject attractive to the student.

Moreover, it is necessary to promote an improvement of nutritional education in Primary Education, where students show attitudes of greater receptivity to new habits and behaviours. In this sense, to carry out this study, we took as a reference the framework of the theory of self-determination proposed by Deci and Ryan [66], which is composed of variables associated with motivation (intrinsic and extrinsic), the autonomy of the individual when deciding what type of behaviour to perform and self-regulation in order to make decisions.

Based on this idea, the main objective of this research is to verify the effect caused after the application of Flipped Classroom and the gamification in the development of motivation, autonomy and self-regulation towards learning through a didactic unit about healthy habits and diet in the Primary Education students. This objective can be stratified in the confirmation of the following research questions:

- R.Q.1. Does experimentation with Flipped Classroom and gamification promote an improvement in the motivation, autonomy and self-regulation of student learning in Primary Education?
- -R.Q.2. What is the degree of statistical dependence between the motivation, autonomy and self-regulation of the learning of the analysed student body?
- R.Q.3. Is the type of educational centre an incident factor in the development of motivation, autonomy and self-regulation of student learning?

## 2. Methodology

A pre-experimental quantitative study is presented in which a methodological design with pre-test and post-test was used to identify the effect caused by the development of these methodologies on the variables of the student body. To this end, the use of different descriptive-inferential statistics was used to accurately describe the responses given by the set of subjects [67,68].

### 2.1. Participants

The teaching experience presented in this document was carried out in four primary schools of different types (two public and two subsidized) in the city of Granada (Spain). The public centres are characterised by being secular centres, financed and managed by the government with the help of the local administrations in each area. They have limited places, and in order to access them, a series of requirements established by the central administration must be met. On the other hand, subsidised centres are private centres, but they are largely subsidised by the central administration. They finance part of their offerings with public subsidies and another with payments from parents [69]. The participants were 202 students in the 6th grade of Primary School who were taking the subject of Natural Sciences (n = 202), 98 belonging to the two public centres and 104 to subsidised centres. The participants were chosen on the basis of a convenience sampling. Specifically, there are 82 boys and 120 girls, aged between 11 and 12 years old (M = 11.75; SD = 0.304). The focus of the study was on the character of the educational centre due to the differences observed in terms of this factor in the measurement of other competence variables shown by other relevant studies [70,71].

With regard to the procedure for recruiting the sample, we first contacted the management team of the centres in order to inform them of the purpose of our work and the duration of its implementation. Subsequently, we proceeded to request permission from the students’ parents to have their consent to distribute the questionnaires to students. Those who agreed to participate signed the informed consent. Some additional socio-demographic data are given in Table 2 below.

The research was approved by the Ethics Committee of the AREA research group (HUM-672) of the Regional Ministry of Economy, Knowledge, Business and Universities of the Andalusian Government (Spain) (Project-0015).

### 2.2. Procedure

**Figure 1:**
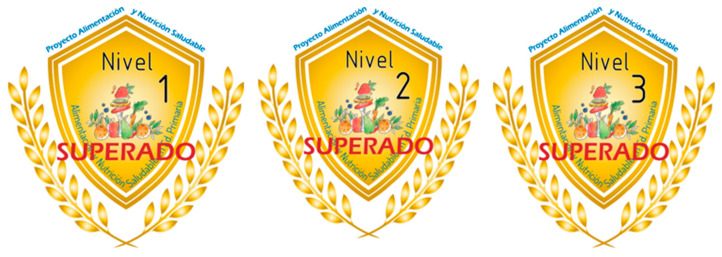
Badges obtained by overcoming challenges, missions and adventures.

As for the development of this investigation, it consisted of four stages:

- Stage 0: Firstly, it was introduced into the conceptual bases of the Flipped Classroom and gamma methodology to the student body. Especially, this phase was aimed at making students aware of the dynamics that would be carried out from that moment on. For the distribution of the Flipped videos, EdPuzzle software was used, which allows all the students to be registered, as well as, at the same time, allowing the teacher the option to check which students watch the videos and which ones do not. Similarly, it offers the option of inserting assessment quizzes in the middle of the video or at the end of the video to check the learning acquired. In this way, each of the students in each class was registered, so that they could access the platform and view the available material. In this case, the day before the class, the teacher uploaded a video that the students had to watch before the class day. In the same way, they completed a questionnaire with questions evaluating the correct viewing of the video. This information was received directly by the teacher.
- Stage 1: The questionnaire was distributed prior to the development of the experience (pre-test).
- Stage 2: From this moment on, the development of a didactic unit on nutritional education and healthy habits was started. It consisted of 7 sessions in which the students would follow the development of the unit through the Flipped Classroom methodology (Table 3). The videos of the Flipped sessions are of our own making. Taking as a reference relevant studies that followed this methodology, it was taken into consideration that the videos provided to the student body should not exceed 10 min in length [43]. Similarly, a greater number of sessions were focused on those contents considered of greater importance, such as the explanation of the types of nutrients, as well as the teaching of daily habits to benefit a healthy lifestyle.
- Stage 3: Once in the classroom, the sessions were framed to carry out activities of a practical nature. One of the final sessions was also dedicated to teaching the importance of knowing oneself and respecting one’s own body and that of others, a transcendental aspect of nutritional education in this period of the students’ age. We proceeded to implement the gamma methodology through the awarding of digital badges, which were given through the LMS Moodle platform, where previously, the contents of the teaching unit (basic, intermediate and advanced), missions and adventures and, finally, levels (Figure 1) had been adapted and sequenced. The challenges corresponded to exercises and activities that students had to complete based on the contents of the teaching unit. Depending on the qualification obtained, the reward was a bronze, silver or gold badge. Obtaining three badges meant that the teaching unit had been passed. Among the most outstanding challenges were bringing in a piece of fruit twice a week to eat during the rest period, the preparation of a weekly record in which the amount of physical exercise done per day was determined, or a research project on properties that are not known in some vegetables.
- Stage 4. Once the experience was completed, the measurement instrument was distributed in order to collect the students’ perceptions and attitudes about this educational experience (post-test). Finally, the results were shared with the students so they could compare their responses before and after this experience.

### 2.3. Instrument

For data collection, we advocated the use of an ad hoc questionnaire that established the focus on the analysis of three specific variables: Motivation (MOT); Autonomy (AUT); and self-regulation (SER). For its configuration, other instruments were taken as references, already validated and of recognized prestige on motivation, self-regulation and learning autonomy (Student Motivation Survey) [72]. The instrument was composed of 16 items distributed as follows: 5-MOT; 5 AUT; 6 SER. As an example, MOT contained items such as “I like the class better when we use technological resources”; AUT contained items such as “I like to watch videos about sport and physical activity on my own”; and SER contained items such as “I make the studio nice by turning it into a game”. As for the nature of the instrument’s measurement, it is a Likert 5 type questionnaire (1: strongly disagree; 2: disagree; 3: neither agree nor disagree; 4: agree; 5: strongly agree).

Likewise, the instrument was submitted to an expert opinion from different universities (University of Seville, University of Malaga and University of Granada), to validate its content. Later, for the validity of the construct, an exploratory factorial analysis was applied with the maximum likelihood extraction method and with Varimax rotation, as well as the calculation of the sphericity coefficient of Barlett (KMO = 0.807; *p* < 0.001). In addition, Cronbach’s Alpha (α = 0.81) was used as an internal consistency index to determine reliability. The optimal result guaranteed the suitability of the instrument to be distributed among the sample of participants.

### 2.4. Data Analysis

The statistical software RStudio in its version 3.6.1 was used for data analysis. Firstly, the common descriptive statistics were applied, which allowed us to approach the description of the students’ attitudes, as well as the differences observed in the pre-test and post-test scores. After this, we analysed the character of the data distribution from the Shapiro–Wilk test, which verified that the trend was not normal (*p* > 0.05). On the other hand, it was verified with the calculation of Levene’s test that the collected answers presented similar variances (*p* > 0.05). Finally, as regards to the independence of the residues from the data, the Durbin–Watson test allowed this condition to be verified and, therefore, guaranteed the future of the analysis. The U-Mann Whitney test was applied in order to determine whether significant differences existed between the averages obtained from the samples of the different groups. Consequently, the correlation between the variables that make up the instrument was analysed through the application of the Pearson test. Finally, it was analysed whether the socio-demographic factor “character of the school” was a determining factor in the development of the constructs through the configuration of multiple linear regression models. Linear regression models were advocated because of the ability to detect the weight and influence of independent variables in the development of numerical constructs [73].

## 3. Results

In the first instance, the calculation of the descriptive statistics established an initial approach to the responses of the participating students (Table 4). Firstly, it was found that the effect of the application of the methodologies led to an improvement in the variables as shown by the post-test results. Thus, the autonomy construct obtained the highest score, followed by self-regulation and motivation. The variability of responses was high in most cases, which may infer the presence of outliers in the data distribution.

After this, and noting the non-parametric nature of the data distribution, the U-Mann Whitney test was calculated, which confirmed the differences between the means of the post-test phase and the pre-test (Table 5).

**Figure 2:**
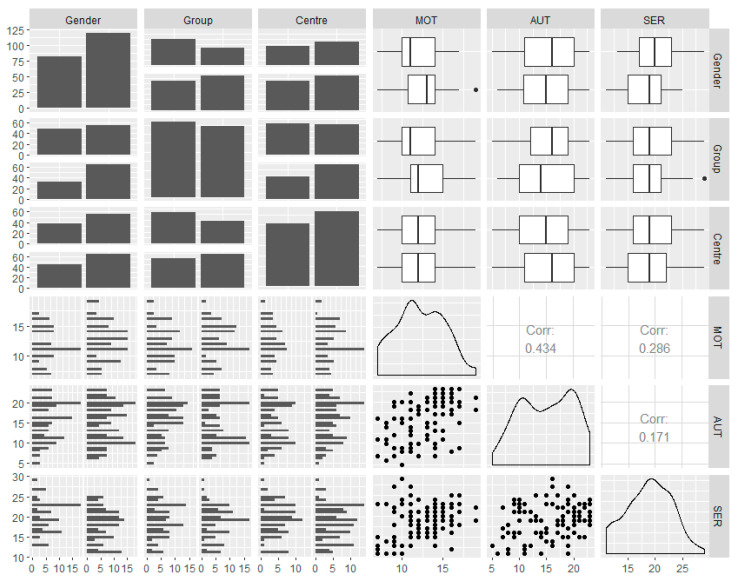
Nature of data distributions and correlation between variables. Note: MOT = motivation; AUT = autonomy; SER = self-regulation.

Therefore, the Pearson correlation test was applied, which allowed us to know the relationships between the study variables (Figure 2). In this case, no very strong correlations between constructs were observed. The moderate link between AUT and MOT and a slight relationship between SER and MOT should be noted.

In order to determine whether the nature of the educational centre was a possible indicator of the development of the research constructs, the multiple regression models were configured, which made it possible to clarify whether the educational centre variable was an incident when presenting better results or not. Thus, three models were configured according to the variables of the study. Those factors marked with an * are those that present an incidence relationship with the construct from which the model was configured. In the first instance, the model elaborated from the MOT variable did not determine that the type of centre was a determining factor for its development (Table 6). However, AUT and SER are presented as strong predictors of this variable. The model was significant (F = 15.78; df = 196; *p* < 0.05) and its adjustment explained almost 27% of the total variance (R^2^ = 0.2689).

In the case of the AUT construct, the model configured did determine that the character of the educational establishment could influence the development of AUT (Table 7). Similarly, a strong link is maintained with MOT. The model was significant (F = 10.68; df = 196; *p* < 0.05) and explained 19% of the total data (R^2^ = 0.1941).

Finally, the model on SER showed that the school could be an influential factor in its development, in addition to MOT (Table 8). The model was significant (F = 8358; df = 196; *p* < 0.05) and explained 18% of the total data (R^2^ = 0.1872).

## 4. Discussion

The advent of technology has led to the arrival of many new methodological approaches that bring a different vision to traditional teaching. Specifically, in the case of nutritional education, the arrival of these pedagogical models has provided dynamism in the teaching/learning process for both teachers and students, providing an alternative way for students to assimilate concepts about nutritional education and a healthy diet that they can apply to their daily routine [56]. In this case, the implementation of Flipped Classroom and gamification through badges has constituted an opportunity to configure a different development of a didactic unit, while allowing students to observe a different way of learning content.

Within this context, the present research aimed to verify how the implementation of the Flipped Classroom and gamification methods affect the healthy habits and diet of Primary Education students. For this purpose, the focus was established on the analysis of the effect on the constructs of motivation, autonomy and self-regulation of learning. Based on this starting point, the findings of the descriptive analysis allowed us to verify that the results of the post-test phase were superior to the pre-tests in the three research constructs. Therefore, we continue on a path of results that allow us to solidify the argument in favour of improving student abilities after the application of active methodologies in the classroom [36,40,47,48,74]. The game-based approach put the students in a scenario of missions, challenges and adventures to be achieved, in such a way that their levels of motivation to achieve the proposed goals were increased. Consequently, the student occupied a leading role in his own teaching/learning process, which caused an increase in his internal and external motivations. These results coincide with previous studies that have applied to this type of methodology in a similar way [60].

Similarly, the implementation of Flipped Classroom gave a role of great responsibility to the student. The development of the first phase of Flipped Classroom, which involved watching videos and filling out questionnaires, was a daily mission that the student had to carry out. This made him aware that part of the development of the teaching unit depended on his own performance. In this way, and in line with previous results, an improvement in the student’s learning autonomy was observed when experimenting with this type of methodology [43]. This idea is especially relevant in the case of the development of healthy lifestyle and dietary habits, since the increase in learning autonomy could lead to a better understanding of this content by the students, promoting a better attitude of responsibility towards the importance of healthy habits and diet. In this way, a direct confrontation is established with the risks of juvenile obesity, child sedentarism and other eating disorders that exist in the young sector today [54].

Consequently, Pearson’s correlation test allowed the verification of proportional relationships between constructs, especially those between AUT and MOT. Based on this link, it is interesting to indicate the existing relationship that other previous studies made about these two variables, referring especially to the increase of autonomy that promotes work through technology, and how this promotes a motivational improvement in the student body [47,48]. In this case, the relationship could be similar, allowing us to establish a reflection of the student of today, his or her relationship with the digital world and how the teacher can take advantage of this characteristic to promote an improvement in their autonomy in learning and, therefore, in their motivation.

Finally, as a result of relevant studies that highlight differences existing in the development of variables around the character of the educational centre, the focus was established on corroborating this premise in this case study. Thus, the configuration of the linear regression models based on the three study constructs determined which variable “Centre” was an influential parameter in the development of AUT and SER. The character of the educational centre implies, as in the case of subsidised centres, greater attention from the centre’s professionals, as well as a very close teacher–student–family relationship, which could influence the student’s levels of autonomy, as previous studies state [68]. Similarly, this factor could be an incident in the self-regulation of the student when it comes to learning. The three models developed were significant, although the variance explained was not considerable. In spite of this, they allow us to establish an approach to what could be the reality of the development of these constructs.

In summary, the results obtained confirm the effectiveness of the application of innovative methods in nutrition education, in the same line that is showed in similar studies [62,63,64,65].

## 5. Conclusions

The arrival of technology in the educational panorama has provoked the arrival of numerous active methodologies that bet on a leading role of the student, who builds his own learning, supported by the figure of the teacher who facilitates this purpose. In the case of nutritional education, it is pertinent to energize this teaching at all educational stages, even more so at the earliest ages, so that students receive this learning in an attractive and dynamic way that predicts future applicability in their daily lives.

Thus, the realization of this research allowed the verification of positive effects that caused the application of the methods Flipped Classroom and gamification through digital badges in students of Primary Education. The variables linked to motivation, autonomy and self-regulation of learning were substantially increased after the educational experience. Similarly, the character of the educational centre was found to be a possible socio-demographic factor that could have an impact on the development of these constructs.

Regarding the limitations of the work, it is argued that a longitudinal methodological design could have been established, which would have tested the effects of these methodologies over a longer period of time and on a regular basis. Likewise, the choice of sampling for convenience as a statistical technique for the selection and scrutiny of the sample of participants was considered to be a restriction presented by this study. On the other hand, as regards future lines of research, it is advocated that the research community should continue to contribute empirical work in which the positive effects of innovative methodologies are guaranteed at different educational stages where nutritional education is worked on, especially at higher stages, where traditional teaching becomes a tedious and complex process to be carried out by both teachers and students. To this end, the promotion of good educational practice will be fundamental in promoting an improvement in nutritional education.

In conclusion, technology provides an amalgam of didactic possibilities to promote an improvement in teaching. Therefore, it is essential that in the field of nutritional education, professionals are dedicated to continue investigating methodologies that allow the development and promotion of quality nutritional education, promoting an improvement in the skills of their students, and disseminating a type of teaching that is related and conducive to the student of today.

## Figures and Tables

**Table 1:** Comparison between traditional methodology, Flipped Classroom and gamification.

Traditional Teaching	Flipped Classroom	Gamification
The role of the student is passive, limited to the role of receiver. The teacher is in charge of transmitting the contents in a theoretical way. After this, a series of activities are sent to the student to be carried out at home. In most cases it is evaluated through a written exam.	The student has two learning phases: (a) a phase outside the classroom, where he or she visualizes training videos and can complete tasks online; (b) in the classroom, he or she prepares practical tasks and projects, and resolves doubts about the material visualized at home. The evaluation is more dynamic, since it contemplates a greater number of indicators (online work, project work, written and oral tests, etc.)	The educational scenario is transformed, elements of play are introduced with a didactic intention. In this case, the game by badges aims to grant recognition of having overcome a task, activity or present a positive behaviour through the granting of these distinctions. These recognitions will be assigned to different parameters of the final evaluation. Therefore, it is an evaluation strategy that is motivating for students.

**Table 2:** Socio-demographic data of the study sample.

	*n*	%
**Centre**		
Public	98	49.02
Private	104	50.98
**Gender**		
Boy	82	40.6
Girl	120	59.4
**Age**		
11	111	54.95
12	91	45.05

**Table 3:** Distribution of the unit’s topics, sessions and hours.

Topics	Development of the Session	Sessions	Nº Videos	Video Duration
1. Types of nutrients	The different types of nutrients are explained: carbohydrates, lipids, proteins, vitamins, water and mineral salts. Exercises are established and daily food and nutrients are related. Similarly, it also refers to the effects that occur if we exceed some of these nutrients.	1	1	7:25 min
2. The balanced diet. The Food Pyramid.	The NAOS food pyramid (set up by the Spanish Ministry of Health) was used. Foods that are at the top are analysed (ice cream, fast food, chocolate, etc.), warned about their excessive consumption, and the number of times per week that foods such as meat, fish, fruits or vegetables should be ingested is described. We work with students to develop a schedule of their daily meals in order to analyse and share them. The aim is to identify who is living a healthy life and who is not.	2	2	9:32 min, 6:30 min
3. Social factors and practices that are detrimental to healthy development	The aim is to make students aware of the importance of preventing harmful habits against health, such as the use of tobacco, alcohol or drugs. Subsequently, awareness activities are established based on this theme.	1	2	6:50 min, 7:15 min
4. Health benefits of physical activity	In collaboration with the Physical Education material, students are presented with various simple exercises to do every day. The objective is to transmit that they should practice at least 60 min of useful physical activity.	2	1	9:30 min
5. Self-concept and bodily respect for others	Activities are presented to raise awareness of the value and acceptance of one’s own physical reality, possibilities and limitations. The focus is on developing values of respect for one’s own body and that of others.	1	1	9:00 min
Total time		7	7	54:82 min

**Table 4:** Descriptive statistics.

Variable	Pre-Test		Post-Test	
	Mean	SD	Mean	SD
MOT	1.94	1.391	2.79	2.737
AUT	2.45	1.783	3.28	0.982
SER	1.71	0.723	2.82	1.89

Note: MOT = motivation; AUT = autonomy; SER = self-regulation.

**Table 5:** Comparison between treatment group averages.

Group	Average Range	Sum of Ranges	U-Mann Whitney	W-Wilcoxon	Z	*p*
Pre-test	91.41	9546	3891	8241	−0.724	0.000
Post-test	105.36	10,957	4695	9546	−0.966	0.000

**Table 6:** Linear regression model for MOT.

	Estimate	Std. Error	t Value	*p* Value	
(Intercept)	3.91870	1.06912	3.665	0.000318	***
Centre	−0.18773	0.36369	−0.516	0.606302	
AUT	0.24808	0.03776	6.570	4.43 × 10^−10^	***
SER	0.19150	0.04531	4.226	3.64 × 10^−5^	***

Note: MOT = motivation; AUT = autonomy; SER = self-regulation; *** = *p* < 0.001.

**Table 7:** Linear regression model for AUT.

	Estimate	Std. Error	t Value	*p* Value	
(Intercept)	6.05672	1.84246	3.287	0.0012	**
Centre	0.81062	0.62054	1.306	0.1930	*
MOT	0.72752	0.11073	6.570	4.43 × 10^−10^	***
SER	0.03263	0.08102	0.403	0.6876	

Note: MOT = motivation; AUT = autonomy; SER = self-regulation; * = *p* < 0.5; ** = *p* < 0.01; *** = *p* < 0.001.

**Table 8:** Linear regression model for SER.

	Estimate	Std. Error	t Value	*p* Value	
(Intercept)	15.11493	1.27125	11.890	2 × 10^−16^	***
Centre	−0.69443	0.54699	−1.270	0.206	*
MOT	0.43614	0.10320	4.226	3.64 × 10^−5^	***
AUT	0.02534	0.06292	0.403	0.688	

Note: MOT = motivation; AUT = autonomy; SER = self-regulation; * = *p* < 0.5; *** = *p* < 0.001.
